# Talquetamab‐Related Dysgeusia in Multiple Myeloma Compared to BCMA‐Targeted Bispecifics and High‐Dose Melphalan

**DOI:** 10.1002/cam4.71401

**Published:** 2025-12-08

**Authors:** Anna Fleischer, Magdalena Roll, Jan H. Frenking, Franziska Panther, Götz Gelbrich, Patrick‐Pascal Strunz, Christine Riedhammer, Jessica Peter, Julia Mersi, Johannes Waldschmidt, Martin Kortüm, Hermann Einsele, Marc‐S. Raab, Imad Maatouk, Leo Rasche

**Affiliations:** ^1^ Department of Internal Medicine II University Hospital Würzburg Würzburg Germany; ^2^ Heidelberg Myeloma Center, Department of Medicine V, University Hospital and Medical Faculty Heidelberg Heidelberg University Heidelberg Germany; ^3^ Institute of Biometry and Epidemiology University Hospital of Würzburg Würzburg Germany

**Keywords:** gustatory dysfunction, immunotherapy, oral complications, quality of life, taste impairment, treatment adherence

## Abstract

**Background:**

Dysgeusia is a side effect of the anti‐GPRC5DxCD3 bispecific antibody talquetamab (TAL), but other myeloma treatments, such as high‐dose melphalan (MEL) with autologous stem cell transplantation (ASCT), are also known to alter taste perception in patients with multiple myeloma (MM). This study investigates the spectrum, prevalence and severity of dysgeusia in patients receiving TAL and MEL and compares the results with anti‐BCMA bispecifics as a control for T‐cell‐engaging therapies.

**Methods:**

Gustatory and olfactory performance was assessed in 87 MM patients divided into three treatment groups: TAL (*n* = 26), MEL/ASCT (*n* = 35), and BCMA bispecifics (*n* = 26). Evaluations included Taste Strips, Sniffin' Sticks Identification Test 16, and comprehensive questionnaires on taste perception, dietary issues, quality of life (QoL), mood, and treatment compliance.

**Results:**

TAL‐treated patients exhibited severe taste impairment, with 96.2% reporting marked declines. Taste alterations were also observed in patients receiving MEL/ASCT and BCMA bispecifics, though these were less pronounced, affecting 62.9% and 30.8% of cases, respectively. Xerostomia incidence was highest in the TAL group. Patients considering discontinuation of TAL (30%) cited taste alterations as the primary reason. MEL was associated with higher incidences of nausea, vomiting, and appetite loss.

**Conclusion:**

TAL‐associated taste disturbances have a major impact on patients and require further investigation and mitigation strategies. Enhanced patient support, proactive monitoring, and targeted interventions are critical to improving the well‐being and adherence of MM patients.

## 1 Introduction

Multiple Myeloma (MM) is a hematologic malignancy characterized by the proliferation of clonal plasma cells in the bone marrow. Despite great advances in therapeutic options, MM remains an incurable disease. Conventional treatments include chemotherapy, such as high‐dose melphalan (MEL) followed by stem cell transplantation, or more targeted therapies, such as anti‐CD38 antibodies, proteasome inhibitors or immunomodulatory agents (IMiDs).

Currently, an era of novel immunotherapies, including T cell‐engaging bispecific antibodies is about to fundamentally alter the treatment algorithm of MM. While these products have remarkable single‐agent activity [1, 2], a number of novel adverse events with potential impact on quality of life (QoL) have been reported. Talquetamab (TAL), a G protein‐coupled receptor (GPRC5D)xCD3 bispecific antibody, has shown promising results in the pivotal MonumenTAL‐1 study, with an overall response rate of ~70% and a median progression‐free survival of 11.2 months in the biweekly dosing arm [3]. However, the expression of GPRC5D in cells within the oral cavity could result in on‐target off‐tumor toxicities, particularly in the form of taste disturbance, a side effect that is often underreported in clinical trials and inadequately managed in clinical routine [1, 4]. Taste perception is a multifaceted sensory function which involves the integration of gustatory and olfactory signals, enabling the identification and enjoyment of distinct taste qualities such as sweet, sour, salty, bitter, and umami. In the context of cancer therapy, taste disturbances can lead to reduced food intake, malnutrition and weight loss. Moreover, the impact of dysgeusia extends beyond physical health, affecting patients' psychological and emotional states and social interactions [5, 6, 7, 8, 9, 10].

The current literature lacks comprehensive studies on taste disturbances induced by newer MM treatments, such as TAL‐ or BCMA‐targeted bispecific antibodies, compared to classical cytotoxic agents with known oral side effects, such as high‐dose MEL. Moreover, most studies do not provide insights into the specific and profound impact of dysgeusia on patients' QoL and treatment adherence.

The primary aim of this study was therefore to systematically characterize, evaluate and compare the extent of dysgeusia among these treatments and to assess their psychosocial impact on MM patients. By identifying the severity and implications of taste disturbances, this study seeks to highlight the need for targeted interventions and support mechanisms to enhance patient care and treatment adherence.

## 2 Methods

We conducted a multicenter, prospective, observational study at the University Hospitals Würzburg and Heidelberg to assess taste and olfactory function, nutrition‐related symptoms, and psychosocial outcomes, in patients with MM undergoing treatment with TAL, MEL/ASCT, or BCMA bispecifics. The study was approved by the local ethics committee (AZ 119/22) and adheres to the tenets of the Declaration of Helsinki. Written informed consent was obtained from all patients.

### 2.1 Study Population

The study included a total of 87 MM patients. Among them, 26 patients had been treated with TAL, 35 had received high‐dose MEL/ASCT and 26 patients had been administered BCMA‐directed bispecifics. BCMA‐directed bispecifics were selected as a comparator to provide a control group based on the shared T‐cell redirection mechanism but targeting a different antigen. This comparison aims to demonstrate that dysgeusia is an on‐target adverse event of GPRC5D, highlighting that it is likely linked to the mechanism of GPRC5D engagement rather than solely due to T‐cell redirection.

Patient demographic and clinical characteristics are detailed in Tables S1 and S2.

The assessments were conducted after the patients had received at least two doses of TAL following the initial step‐up doses. The study included patients from cycle 1 to cycle 10, with 61.5% of the patients being in either cycle one or two. The objective was to gather data from as many TAL patients as possible, regardless of any previously indicated symptoms related to dysgeusia.

### 2.2 Taste Strips Assessment

Test strips were purchased at Burghart Messtechnik GmbH; Uetersener Straße 6; D‐25488 Holm. Participants were presented with blinded filter paper strips impregnated with various taste substances representing the five primary taste qualities: sweet, sour, salty, bitter, and umami, as well as negative controls. Participants placed each strip on their tongue and indicated the perceived taste quality. Varying concentrations of taste substances on the strips allowed for the determination of taste sensitivity thresholds for each taste quality [11]. This quantitative assessment provided data on taste perception and sensitivity among participants.

### 2.3 Sniffin' Sticks Assessment

Sniffin' Sticks were purchased at Burghart Messtechnik GmbH; Uetersener Straße 6; D‐25488 Holm. Participants were presented with a series of blinded pen‐like devices containing different odorants and were asked to identify each scent. The test encompassed a range of common odorants representing various categories, such as fruits, spices, and household items. Participants' responses were scored based on the accuracy of odor identification. This objective assessment enabled the detection of changes in smell sensitivity or discrimination abilities associated with MM treatments [12].

### 2.4 Oral Cavity Inspection

An oral cavity inspection was conducted to identify any visible abnormalities or conditions affecting oral health that may contribute to taste disturbances or other symptoms reported by participants.

### 2.5 Assessment of Quality of Life, Depression, and Anxiety

Quality of life was evaluated using the validated EORTC QLQ‐C30 (version 3.0) [13], while the validated PHQ‐4 [14] was utilized to assess depression and anxiety.

### 2.6 Assessment of Patient‐Reported Treatment Side Effects

Patient‐reported treatment side effects were captured through a mixed‐methods questionnaire. This instrument focused on aspects such as nutritional status, oropharyngeal symptoms, baseline characteristics, therapy adherence, the social impact of MM treatment, and patients' receptiveness to engaging in support services.

### 2.7 Statistical Analysis

Data were analyzed using descriptive statistics to summarize baseline characteristics and sensory assessment results. Comparative analyses were conducted using ANOVA for continuous variables and chi‐square tests for categorical variables to evaluate differences between treatment groups. Correlations between sensory assessment outcomes and psychosocial measures were examined using Pearson's correlation coefficient. A *p*‐value of < 0.05 was considered statistically significant.

## 3 Results

### 3.1 Taste Disturbances

**FIGURE 1:**
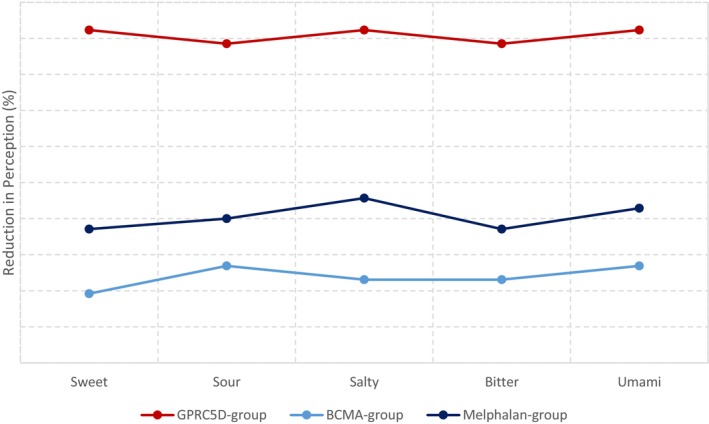
Subjectively perceived reduction in perception of flavors.

We enrolled 87 MM patients divided into three treatment arms: 26 patients receiving TAL (GPRC5D group), 35 patients receiving high‐dose MEL, and 26 patients receiving BCMA bispecifics (teclistamab or elranatamab) (Tables S1 and S2). The vast majority of patients treated with TAL (96.2%) reported significant impairments in overall taste performance, which were objectively confirmed in 92.3% of cases through blinded taste strips testing. Patients receiving MEL and BCMA bispecifics also exhibited taste disturbances, albeit less pronounced, namely in 62.9% and 30.8% of cases, respectively. The subjective reduction in the perception of distinct flavors was more notable in the GPRC5D group for all flavor types (sweet, sour, salty, bitter, and umami), with all *p*‐values indicating statistical significance (*p* < 0.05). Not only the prevalence but also the severity of taste disturbances was higher in the group of patients treated with TAL compared to the other groups (Figure 1 and Table S3).

Interestingly, objective testing showed no significant differences in olfactory perception between patients receiving TAL, MEL, or BCMA bispecifics (Table S4). In summary, TAL caused the most severe taste abnormalities, whereas olfactory perception was not altered by any of the three treatments.

Given the impact of taste disturbances, there was a high demand for symptom‐specific support services among TAL patients, with 76.9% expressing a strong desire for the development of a program to improve their taste experience.

### 3.2 Nutrition‐Related Symptoms

**FIGURE 2:**
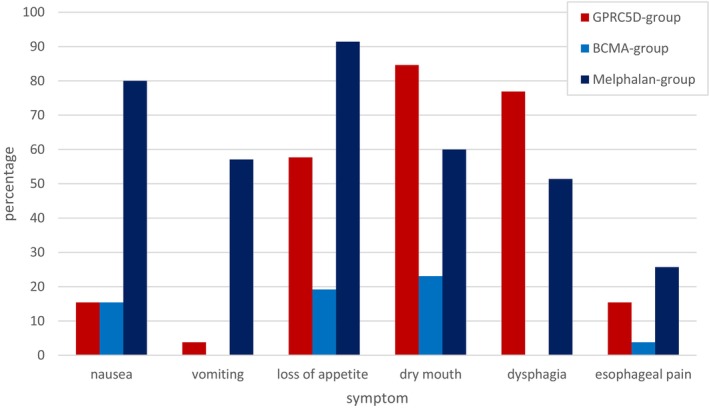
Nutrition‐related symptoms.

**FIGURE 3:**
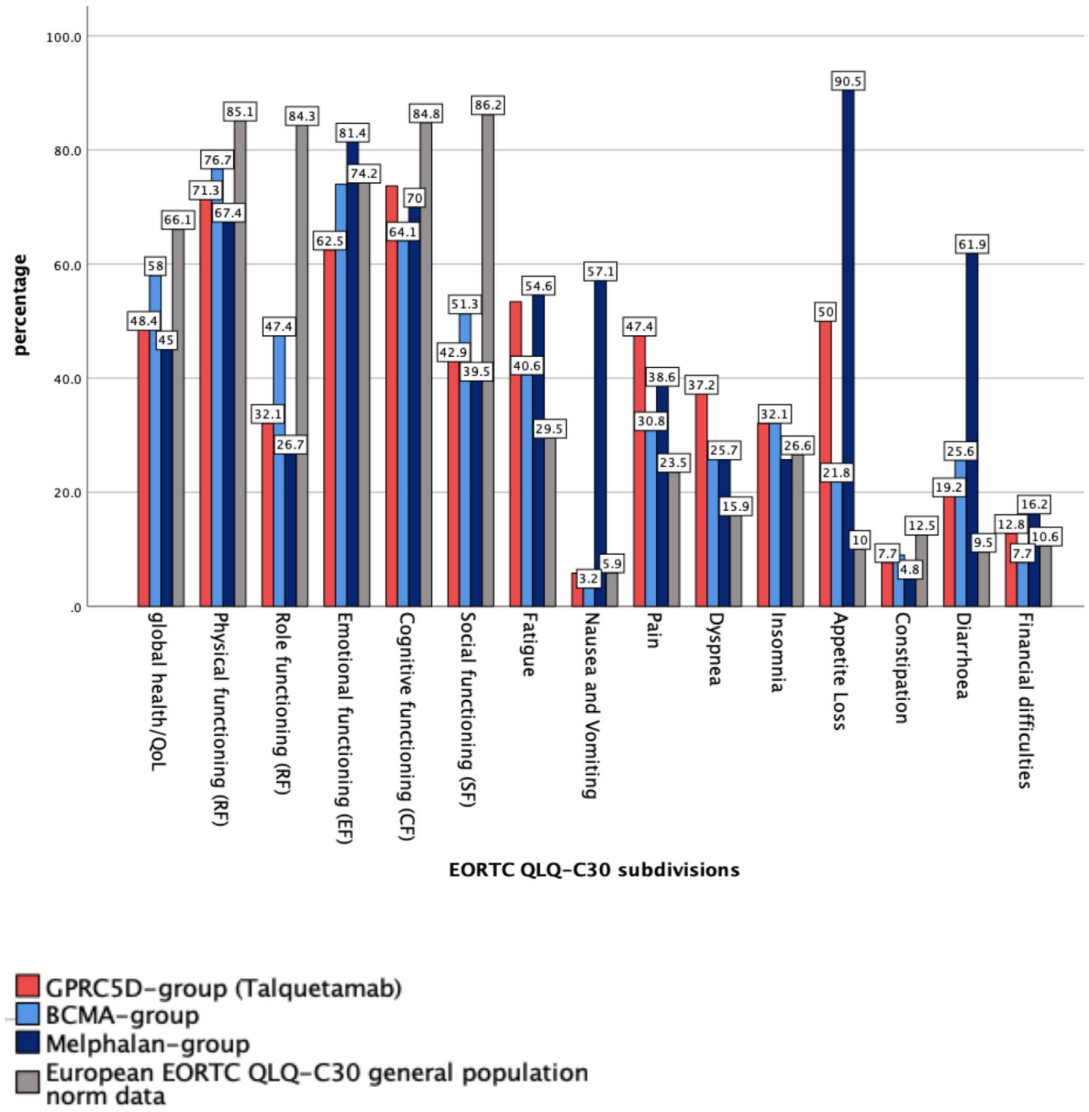
Analysis of the EORTC QLQ‐C30 health score, functioning scores and symptom scores. The European EORTC QLQ‐C30 general population norm data is based on 15,386 persons across 13 European countries, Canada, and the United States [15].

**FIGURE 4:**
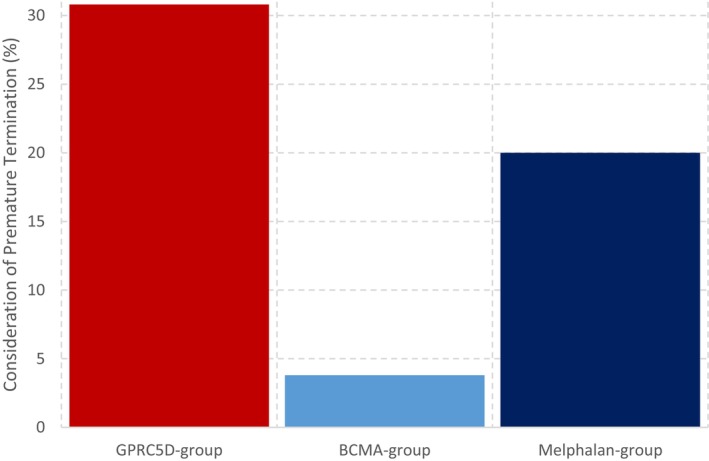
Consideration of premature termination of treatment.

Loss of appetite was most frequently reported in the MEL/ASCT group (91.4%) compared to the GPRC5D group (57.7%) and the BCMA group (19.2%, *p* < 0.001) (Figure 2 and Table S5). Chi‐squared tests of independence show that there was an association between the symptoms of nausea/vomiting and loss of appetite in MEL‐treated patients (*p* < 0.001). In line, severe nausea and severe vomiting had their highest occurrence in the MEL group (48.6%, *p* < 0.001 and 28.6%, *p* = 0.001, respectively), compared to 3.8% of severe nausea in the GPRC5D group, 0% severe nausea in the BCMA group, and 0% of severe vomiting in both the GPRC5D group and the BCMA group (Figures 3 and 4).

Patients in the TAL group experienced the highest average weight loss since the beginning of therapy at 3.3 kg (SD 4.4, range 0–15 kg), followed by the MEL group with an average loss of 2.6 kg (SD 2.5, range 0–10 kg). In contrast, patients in the BCMA group showed a minimal average weight loss of 0.54 kg (SD 1.4, range 0–5 kg).

The corresponding average percentage weight loss, calculated as [weight lost (kg)/current weight (kg) × 100], further highlights these differences: TAL patients showed an average percentage weight loss of 4.4% (SD 5.8, range 0%–19.1%), MEL patients had an average percentage loss of 2.8% (SD 3.8, range −9.9% to 11.5%), and BCMA patients exhibited a slight average weight change of −0.21% (SD 3.5, range −10.9% to 7.2%), indicating occasional slight weight gain in this group. Of note, these numbers represent a snapshot in time, as treatment duration was heterogeneous among patients and no follow‐up data were collected.

Dry mouth, however, was most common in the GPRC5D group (84.6%, *p* = 0.02) compared to the MEL group (60.0%) and the BCMA group (23.1%) (Figure 2). A correlation (*p* < 0.05) was observed between dry mouth and subjective taste impairment for sweet, sour, bitter, salty and umami tastes in TAL‐treated patients. Consistent with dry mouth, dysphagia was frequently reported in the GPRC5D group (76.9%, *p* < 0.001), compared to 51.4% in the MEL group and 0% in the BCMA group (Figure 2). There was also a significant correlation between dysphagia and dry mouth (*p* < 0.05) and between dysgeusia and dysphagia (*p* < 0.05) in the TAL group.

Examination of the oral cavity confirmed that dry mouth was most prevalent in the GPRC5D group (30.8% severe cases, *p* < 0.001). However, open wounds in the oral cavity were most frequently observed in the MEL group (14.3% moderate cases, *p* = 0.02). Additionally, 34.6% of patients in the BCMA group reported experiencing a recent or current infection or cold, which may have influenced taste and nutrition‐related symptoms. Table S6 shows significant differences in the results of the oral cavity examination between the treatment groups.

### 3.3 Comorbidities Affecting Taste Or Olfactory Perception

To identify potential confounding factors, patients were queried about comorbidities that could potentially affect taste or olfactory perception (Table S7). There were no statistically significant differences in the prevalence of preliminary diagnoses, including a history of smoking, when comparing the GPRC5D group, the MEL group, and the BCMA group. Importantly, no differences were noted in the prevalence of peripheral neuropathy among the three groups.

### 3.4 Quality of Life (QoL)

Due to the potential impact of dysgeusia on physical and emotional well‐being, we next compared QoL measurements between the three groups. Most patients in each group provided intermediate ratings, with only a small proportion rating their QoL as “very bad” or “excellent”.

When focusing on health scores, the MEL group had the lowest mean scores (45.0, *p* < 0.05), reflecting the greatest overall health burden of this therapy. The GPRC5D group exhibited a mean health score of 48.4, which was therefore significantly lower than the general population norm, indicating poorer overall health perception, while the BCMA group showed intermediate health scores, with a mean score of 58, still below the general population norm but higher than those of the GPRC5D group (Figure 3).

The functioning scores for physical, role, cognitive, and social functioning were lower in all patient groups compared to the general population norm. The GPRC5D group had the lowest scores in emotional functioning. The BCMA group scored higher in physical, role and social functioning compared to the other patient groups, while the MEL group exhibited particularly low physical, role and social functioning scores (Figure 3). The differences were not statistically significant.

Symptom scores indicated a greater symptom burden in all patient groups compared to the general population norm.

### 3.5 Psychological Distress

The PHQ‐4 questionnaire measures overall psychological distress, including anxiety and depression. The distribution of PHQ‐4 scores across treatment groups indicated higher overall distress in the GPRC5D group, with elevated levels of anxiety and depression compared to the MEL and BCMA groups. A chi‐square test for the total PHQ‐4 score categories revealed a notable difference among the groups (*p* = 0.015), suggesting that patients in the GPRC5D group experience greater psychological burden.

### 3.6 Treatment Adherence and Patient Considerations

TAL patients were the largest group to consider discontinuing treatment prematurely, accounting for 30% of those surveyed (Figure 4). When asked about the factors influencing their consideration, respondents gave heterogeneous reasons. However, the primary reason cited by all patients contemplating discontinuation due to side effects was a change in taste or a complete loss of taste. Patients expressed discontent with taste disturbances, describing the experience of eating during TAL treatment as burdensome. Anxiety over weight loss due to taste alterations and diminished enjoyment of meals was a concern (19.2%). Some patients inquired about the possibility of reducing the TAL dose to mitigate this side effect. Additionally, 46.2% of patients reported reduced pleasure in consuming specific foods, such as fibrous meat dishes or roasts, which were challenging to swallow due to dry mouth. They conveyed that, prior to TAL therapy, food had been an important source of self‐reward and joy. The onset of taste disturbances resulted in a profound loss of this source of pleasure for 61.5% of patients, leading to frustration when familiar taste perceptions during meals remained unfulfilled. Patients reported that the loss of taste had been a constant concern since the initiation of TAL therapy. Other patients (15.4%) disclosed that, post‐TAL therapy, they had been dining out less frequently with friends, resulting in a sense of loss in this form of social bonding.

In addition to taste alterations, other GPRC5D‐related toxicities such as skin‐related symptoms, including fingernail loss and persistent itching, were reported as reasons for considering discontinuing TAL therapy. Furthermore, physical, cognitive, and emotional fatigue were factors prompting patients to contemplate discontinuation.

Impairments in family life and social activities due to treatment symptoms were observed across all patient groups; however, no statistically significant differences were found among the groups (Table S8).

Respondents emphasized that side effects would need to be extremely severe for them to consider discontinuing therapy, given the limited availability of alternative treatments. This threshold indicates the patients' determination to continue with the therapy despite the adverse effects, underscoring the challenges they face in managing the side effects while maintaining treatment adherence.

## 4 Discussion

This study provides first insights into the nuanced impact of MM treatments on gustatory and related symptoms, underscoring the need for tailored interventions to enhance patient nutritional status and overall well‐being. Our findings highlight the profound taste disturbances associated with TAL and the urgent need for further research and mitigation strategies. Additionally, the study reveals previously underreported dysgeusia in patients receiving MEL and BCMA‐targeted bispecifics. In BCMA‐treated patients, this dysgeusia appears to be primarily linked to sinusitis and respiratory infections.

Our findings show that patients receiving TAL experienced a decline in taste function, with the majority reporting severe taste impairment. This is consistent with previous research indicating that TAL treatment can lead to marked gustatory dysfunctions [1, 16, 17, 18]. Patients vividly expressed the emotional toll of these taste disturbances, including feelings of frustration and disappointment, highlighting the profound impact on their daily lives and enjoyment of food. These results align with studies showing how limitations in taste perception can significantly affect mood and QoL [19, 20, 21]. Furthermore, the study identified significant differences regarding QoL impairment among the investigated treatment groups, with TAL recipients experiencing the most pronounced dysgeusia‐related impairment. We acknowledge that TAL patients tend to be more heavily pretreated than patients in the BCMA group, which may also explain some of the differences in QoL.

The reported reduction in meal enjoyment highlights the broader impact of taste disturbances on nutritional intake and overall physical well‐being. Previous studies have demonstrated that taste impairments negatively affect the well‐being and outcomes of cancer patients, often leading to malnutrition and vitamin deficiencies [22, 23, 24]. Importantly, patients' inquiries about potential solutions and their willingness to explore adaptive strategies highlight the critical role of patient engagement in the treatment process. Addressing taste alterations and related symptoms is essential not only for improving patient comfort but also for mitigating potential nutritional deficiencies and enhancing treatment adherence.

The study emphasizes the multifaceted side effects associated with MM treatments. Notably, patients in the GPRC5D group experienced higher incidences of dry mouth and dysphagia, which further contributed to their consideration of treatment discontinuation. This finding is consistent with broader literature on the side effects of novel MM therapies, where dry mouth and related symptoms are significant concerns [25, 26, 27]. The association between GPRC5D expression and oral side effects, particularly in tissues like the salivary glands, suggests a possible mechanism behind the frequent reports of dry mouth in patients undergoing GPRC5D‐targeted therapies. Although GPRC5D's role in these tissues remains incompletely understood, its documented presence in the oral cavity points toward a localized on‐target off‐tumor effect that may underlie these adverse reactions. MEL‐induced nausea and vomiting were more common compared to TAL and BCMA bispecifics, which is consistent with MEL's known classification as an emetogenic chemotherapy agent. This effect is further intensified by the high dosage and the myeloablative nature of MEL, which contribute significantly to the severity of these side effects [28, 29, 30].

Interestingly, objective olfactory testing did not reveal significant differences in olfactory perception among patients receiving TAL, MEL, or BCMA bispecifics. Given the dominant role of olfaction in flavor perception and food enjoyment [31, 32], this finding highlights a potential area for therapeutic intervention through olfactory enhancement strategies.

Notably, all patient groups reported lower functioning and higher symptom scores compared to general population norms, indicating a substantial burden of disease and treatment on their overall QoL. This is consistent with previous studies that highlight the significant impact of MM and its treatments on patients' physical, psychological, and social functioning [33].

Patients' considerations for discontinuing TAL therapy were primarily driven by taste alterations, highlighting the significant subjective burden of dysgeusia. This aligns with research indicating that taste disturbances are major factors affecting patients' QoL and treatment adherence in oncology [34, 35]. Skin‐related symptoms and fatigue were also important contributors to patients' contemplation of discontinuing therapy, consistent with previous studies [36]. Although taste alterations led many patients to consider stopping treatment, the actual discontinuation rates due to dysgeusia in the MonumenTAL‐1 study were low (only three patients) [3]. This discrepancy might be discussed in relation to the promising results of TAL and the strong determination of patients to live and overcome their disease. In our study, many patients also expressed hope that dysgeusia would improve over time, frequently inquiring if there was data to support this improvement. The Phase 1/2 MonumenTAL‐1 Study also noted initial weight loss in patients undergoing TAL therapy. However, as treatment progressed, weight generally stabilized and even improved, suggesting a similar trend might be expected for patients in this study. This observation may provide reassurance to patients and clinicians about the manageable nature of this side effect over the longer term.

The absence of differences in the prevalence of peripheral neuropathy among the treatment groups suggests that the observed taste and oral health variations are more likely target‐specific rather than neuropathic in origin.

Studies have shown that chronic symptoms and side effects can significantly impact mental health outcomes in cancer patients [37]. The higher levels of psychological distress, anxiety, and depression reported in the GPRC5D group highlight the need for integrated psychosocial support in MM care. However, due to study design, a causal relationship between taste disturbances and depression cannot be established. An alternative explanation may be that patients in the TAL group were at more advanced stages of the disease compared to those in the other groups.

Impairments in both family life and social activities due to treatment‐related symptoms were reported across all groups. This aligns with existing literature on the psychosocial challenges faced by cancer patients, including social isolation, changes in family dynamics, and reduced participation in social activities [38, 39]. Addressing these challenges through comprehensive support services is crucial to improving the overall well‐being of MM patients. For healthcare providers, it is essential to proactively monitor and manage taste disturbances in patients undergoing TAL and other MM therapies. Implementing tailored nutritional support and intervention strategies can mitigate the negative impact on patients' nutritional intake and QoL. Furthermore, integrating psychosocial support into treatment plans can help address the emotional and social challenges associated with taste disturbances and other side effects. This holistic approach can enhance patient satisfaction, treatment adherence, and overall outcomes.

This study has several limitations. First, the relatively small sample size may limit the generalizability of the findings. Future research should involve multicenter studies with larger cohorts to validate these results. Additionally, while significant associations between treatment types and taste disturbances were identified, the observational nature of the study precludes establishing causality. It is also important to consider the proportion of patients in the TAL and BCMA bispecific groups who had undergone prior treatment with high‐dose MEL and ASCT. In contrast, MEL‐treated patients were primarily in their first treatment line and thus more treatment‐naive. This discrepancy may introduce a selection bias, as patients in the TAL and BCMA groups may have experienced cumulative treatment effects. The forthcoming TALISMAN study, which will specifically explore interventions for dysgeusia and taste alterations, is expected to provide further insights and may address some of the limitations noted in our current findings.

Nevertheless, the findings of our study highlight the substantial impact of TAL‐related dysgeusia on patients with MM and underscore the need for targeted supportive measures to improve their QoL and treatment adherence. By systematically characterizing taste disturbances across different treatments, our study contributes valuable insights that may inform clinical strategies and future research, ultimately enhancing patient care in this challenging area.

## Author Contributions

**Anna Fleischer:** conceptualization (lead), data curation (lead), formal analysis (lead), investigation (supporting), methodology (lead), project administration (equal), supervision (equal), validation (equal), visualization (equal), writing – original draft (lead), writing – review and editing (lead). **Magdalena Roll:** formal analysis (equal), investigation (lead), visualization (equal), writing – original draft (equal). **Jan H. Frenking:** writing – original draft (equal). **Franziska Panther:** investigation (equal), project administration (equal), resources (equal), writing – original draft (equal). **Götz Gelbrich:** writing – original draft (equal). **Patrick‐Pascal Strunz:** writing – original draft (equal). **Christine Riedhammer:** writing – original draft (equal). **Jessica Peter:** writing – original draft (equal). **Julia Mersi:** writing – original draft (equal). **Johannes Waldschmidt:** writing – original draft (equal). **Martin Kortüm:** writing – original draft (equal). **Hermann Einsele:** resources (equal), supervision (equal), writing – original draft (equal). **Marc‐S. Raab:** investigation (equal), resources (equal), writing – original draft (equal). **Imad Maatouk:** conceptualization (equal), resources (equal), supervision (equal), writing – original draft (equal). **Leo Rasche:** conceptualization (lead), data curation (equal), formal analysis (equal), methodology (equal), project administration (lead), resources (lead), supervision (lead), validation (equal), visualization (equal), writing – original draft (equal), writing – review and editing (equal).

## Conflicts of Interest

The authors declare no conflicts of interest.

## Supporting information


**Table S1–S8.** cam471401‐sup‐0001‐TablesS1‐S8.docx.

## Data Availability

The data supporting the findings of this study are available from the corresponding author upon reasonable request.
