# Supplementary material for: Talquetamab‐Related Dysgeusia in Multiple Myeloma Compared to BCMA‐Targeted Bispecifics and High‐Dose Melphalan
Source: Cancer Med. 2025 Dec 8;14(23):e71401. doi: 10.1002/cam4.71401 (PMC12685487; doi:10.1002/cam4.71401)
Supplement: Supplementary file 1 — Table S1–S8. cam471401‐sup‐0001‐TablesS1‐S8.docx. [file CAM4-14-e71401-s001.docx]

**Supplementary Table 1: Demographic Characteristics**

| **Characteristic** | **GPRC5D-group (Talquetamab)** | **BCMA-group (Teclistamab, Elranatamab)** | **Melphalan-group** |
| --- | --- | --- | --- |
| **age, years, median, range** | 64.0, 48-85 | 64.5, 52-79 | 62.0, 49-75 |
| **gender** | | | |
| **male, n (%)** | 18 (69.2) | 12 (46.2) | 22 (62.9) |
| **female, n (%)** | 8 (30.8) | 14 (53.8) | 13 (37.1) |

**Supplementary Table 2: Clinical Characteristics**

| **Characteristic** | **GPRC5D-group (Talquetamab)** | **BCMA-group (Teclistamab, Elranatamab)** | **Melphalan-group** |
| --- | --- | --- | --- |
| **ISS-stage of MM** | | | |
| **stage 1, n (%)** | 2 (9.5) | 12 (60.0) | 11 (36.7) |
| **stage 2, n (%)** | 8 (38.1) | 5 (25.0) | 11 (36.7) |
| **stage 3, n (%)** | 11 (52.4) | 3 (15.0) | 8 (26.7) |
| **CRAB-criteria** | | | |
| **hypercalcemia, n (%)** | 3 (11.5) | 2 (7.7) | 6 (17.1) |
| **renal insufficiency, n (%)** | 8 (30.8) | 3 (11.5) | 8 (22.9) |
| **anaemia, n (%)** | 20 (76.9) | 15 (57.7) | 22 (62.9) |
| **osteolytic lesions, n (%)** | 21 (80.8) | 19 (73.1) | 24 (68.6) |
| **Duration of cancer treatment since diagnosis (years), mean (SD), range** | 7.7, 0.9-18.3 | 6.6,  0.8-18.3 | 15.0, 0.6-18.4 |

**Supplementary Table 3: Objective assessment of gustatory perception:**

|  | **GPRC5D-group (Talquetamab)** | **BCMA-group** | **Melphalan-group** |
| --- | --- | --- | --- |
| **Total score of gustatory assessment (Taste strips): mean (SD), range (results without umami*)** | 2.9 (2.9), 0-11 | 9.0 (3.1), 1-14 | 7.7 (3.4), 2-14 |

***** A score of 9 or higher is considered normogeusia, with the maximum possible score being 16 (Instructions for Taste Strips, Burghart Messtechnik GmbH).

**Supplementary Table 4: Objective Assessment of Smell Perception („Sniffin‘ Sticks“)**

| **Characteristic** | **GPRC5D-group (Talquetamab)** | **BCMA-group (Teclistamab, Elranatamab)** | **Melphalan-group** |
| --- | --- | --- | --- |
| **sum of correctly identified fragrances, mean (SD), (range)** | 11.46 (2.9),  (4-15) | 10.24 (3.5),  (4-15) | 11.94 (2.9),  (3-16) |

**Supplementary Table 5: Nutrition-Related Symptoms**

| **Symptom** | **GPRC5D-group (Talquetamab)** | **BCMA-group (Teclistamab, Elranatamab)** | **Melphalan-group** |
| --- | --- | --- | --- |
| **loss of appetite, n (%)** | 15 (57.7) | 5 (19.2) | 32 (91.4) |
| **dry mouth, n (%)** | 22 (84.6) | 6 (23.1) | 21 (60.0) |
| **esophageal pain, n (%)** | 4 (15.4) | 1 (3.8) | 9 (25.7) |
| **dysphagia, n (%)** | 20 (76.9) | 0 (0) | 18 (51.4) |
| **nausea, n (%)** | | | |
| **severe** | 1 (3.8) | 0 (0) | 17 (48.6) |
| **moderate** | 1 (3.8) | 1 (3.8) | 10 (28.6) |
| **little** | 2 (7.7) | 3 (11.5) | 1 (2.9) |
| **no** | 22 (84.6) | 22 (84.6) | 7 (20.0) |
| **vomiting, n (%)** | | | |
| **severe** | 0 (0) | 0 (0) | 10 (28.6) |
| **moderate** | 0 (0) | 0 (0) | 8 (22.9) |
| **little** | 1 (3.8) | 0 (0) | 2 (5.7) |
| **no** | 25 (96.2) | 26 (100) | 15 (42.9) |
| **body mass index, kg/m^2^, mean (SD), range** | 23.7 (3.0), 19.2-29.9 | 24.9 (5.0), 18.5-43.3 | 26.8 (3.4), 21.1-33.2 |
| **average weight loss since beginning of therapy, kg, mean (SD), range.** | 3.3 (4.4), 0-15 | 0.54 (1.4), 0-5 | 2.6 (2.5), 0-10 |
| **subjectively perceived reduction in perception of distinct flavors, n (%)** | | | |
| **subjectively perceived reduction in perception of sweet flavors** | 24 (92.3) | 5 (19.2) | 13 (37.1) |
| **subjectively perceived reduction in perception of sour flavors** | 23 (88.5) | 7 (26.9) | 14 (40.0) |
| **subjectively perceived reduction in perception of salty flavors** | 24 (92.3) | 6 (23.1) | 16 (45.7) |
| **subjectively perceived reduction in perception of bitter flavors** | 23 (88.5) | 6 (23.1) | 13 (37.1) |
| **subjectively perceived reduction in perception of umami flavors** | 24 (92.3) | 7 (26.9) | 15 (42.9) |

**Supplementary Table 6: Oral Cavity Inspection**

| **Characteristic** | **GPRC5D-group (talquetamab)** | **BCMA-group (teclistamab, elranatamab)** | **Melphalan-group** |
| --- | --- | --- | --- |
| **dry mouth, n (%)** | | | |
| **no** | 2 (7.7) | 21 (80.8) | 18 (51.4) |
| **slight** | 6 (23.1) | 2 (7.7) | 11 (31.4) |
| **moderate** | 10 (38.5) | 3 (11.5) | 5 (14.3) |
| **severe** | 8 (30.8) | 0 (0) | 1 (2.9) |
| **open wounds, n (%)** | | | |
| **no** | 24 (92.3) | 24 (92.3) | 27 (77.1) |
| **slight** | 2 (7.7) | 2 (7.7) | 3 (8.6) |
| **moderate** | 0 (0) | 0 (0) | 5 (14.3) |
| **severe** | 0 (0) | 0 (0) | 0 (0) |
| **oral mycosis, n (%)** | | | |
| **no** | 25 (96.2) | 26 (100) | 34 (97.1) |
| **slight** | 1 (3.8) | 0 (0) | 0 (0) |
| **moderate** | 0 (0) | 0 (0) | 1 (2.9) |
| **severe** | 0 (0) | 0 (0) | 0 (0) |
| **damaged teeth, n (%)** | | | |
| **no** | 18 (69.2) | 14 (53.8) | 28 (80.0) |
| **slight** | 5 (19.2) | 4 (15.4) | 4 (11.4) |
| **moderate** | 3 (11.5) | 6 (23.1) | 2 (5.7) |
| **severe** | 0 (0) | 2 (7.7) | 1 (2.9) |
| **coated tongue, n (%)** | | | |
| **no** | 15 (57.7) | 5 (19.2) | 13 (37.1) |
| **slight** | 7 (26.9) | 12 (46.2) | 13 (37.1) |
| **moderate** | 4 (15.4) | 4 (15.4) | 5 (14.2) |
| **severe** | 0 (0) | 5 (19.2) | 4 (11.4) |
| **red throat, n (%)** | | | |
| **no** | 22 (84.6) | 25 (96.2) | 32 (91.4) |
| **slight** | 4 (15.4) | 1 (3.8) | 2 (5.7) |
| **moderate** | 0 (0) | 0 (0) | 1 (2.9) |
| **severe** | 0 (0) | 0 (0) | 0 (0) |

**Supplementary Table 7: Analysis of the EORTC QLQ-C30 Questionnaire Functional Scale Findings by Study Group, mean scores (SD)**

| **Measure** | **GPRC5D-group (talquetamab)** | **BCMA-group (teclistamab, elranatamab)** | **Melphalan-group** |
| --- | --- | --- | --- |
| **Health Scores** | | | |
| **Health Score** | 48.4 (22.5) | 58.0 (16.4) | 45.0 (20.5) |
| **Functioning Scores** | | | |
| **Physical Functioning** | 71.3 (22.9) | 76.7 (13.1) | 67.4 (23.2) |
| **Role Functioning** | 32.1 (39.4) | 47.4 (42.1) | 26.7 (31.1) |
| **Emotional Functioning** | 62.5 (27.8) | 74.0 (22.3) | 81.4 (17.0) |
| **Cognitive Functioning** | 73.7 (30.2) | 64.1 (28.9) | 70.0 (28.2) |
| **Social Functioning** | 42.9 (42.2) | 51.3 (39.4) | 39.5 (38.8) |
| **Symptom Scores** | | | |
| **Fatigue** | 53.4 (27.9) | 40.6 (22.6) | 54.6 (25.6) |
| **Nausea and Vomiting** | 5.8 (13.2) | 3.2 (8.2) | 57.1 (35.8) |
| **Pain** | 47.4 (39.4) | 30.8 (32.6) | 38.6 (38.1) |
| **Dyspnea** | 37.2 (38.1) | 26.9 (29.8) | 25.7 (29.2) |
| **Insomnia** | 32.1 (38.3) | 32.1 (34.6) | 25.7 (29.2) |
| **Appetite Loss** | 50.0 (47.4) | 21.8 (36.4) | 90.5 (26.3) |
| **Constipation** | 7.7 (19.6) | 9.0 (20.1) | 4.8 (16.5) |
| **Diarrhoea** | 19.2 (30.1) | 25.6 (34.4) | 61.9 (39.7) |
| **Financial Difficulties** | 12.8 (28.4) | 7.7 (23.7) | 16.2 (29.6) |

**Supplementary Table 8: Subjectively Perceived Impairment in Family Life and Social Activities by Symptoms of Cancer Treatments**

| **Group** | **GPRC5D (Talquetamab)** | **BCMA (Teclistamab, Elranatamab)** | **Melphalan** |
| --- | --- | --- | --- |
| **Subjectively perceived impairment of family life by symptoms of cancer treatment, n (%)** | 18 (69.2) | 17 (56.4) | 27 (77.1) |
| **Subjectively perceived impairment of get-togethers or joint activities with other people by symptoms of cancer treatment, n (%)** | 18 (69.2) | 18 (69.2) | 27 (77.1) |
